# A History of the Epidemic Cholera, Which Prevailed, during the Summer of 1852, at Chambersburg, Pa.

**Published:** 1852-12

**Authors:** A. H. Senseny


### *A History of the Epidemic Cholera, which prevailed, during the summer of* 1852, *at Chambersburg, Pa.* By A. H. Senseny, M. D.

It is proposed, in the present communication, to give a brief history of the *epidemic* which prevailed in Chambersburg and its vicinity from the 15th of July, 1852, until the 31st of October of the same year. The unusual length of time the disease prevailed, and its extreme virulence of character, seem to demand that all the facts connected with its visitation and prevalence be placed upon record, and to justify the present undertaking.

Cholera, as an epidemic, or in a sporadic form, has made its appearance so frequently and so extensively in many parts of our country, that almost every physician of any experience is more or less acquainted with its nature; but as a great difference of opinion exists among physicians as to the predisposing cause of the disease, and the manner in which it is transmitted from place to place, together with other particulars connected with its appearance in one spot in preference to another, a few preliminary observations may not be deemed irrelevant or improper.

In the first place, I would give the reader an account of the weather during a few months immediately preceding the breaking out of the cholera, and also touch upon the character of the diseases occurring during the same period. Throughout the month of March there was little clear weather; it either snowed, rained, or was cloudy. The same may be said of the month of April. The principal diseases throughout these two months were pneumonic affections, inflammatory rheumatism, and some scattered cases of typhoid fever. During June, the weather was very unstable—only seven clear days intermingled with those that were either showery, cloudy or windy. Diarrhœa, and some few sporadic cases of dysentery, made their appearance during this month. From the 1st to the 13th of July it was dry, very hot, and no rain. On the 15th of this month we had the first case of cholera; the weather was then hazy and warm. From this period until the first of October, clouds, showers and warmth predominated. As I may observe in another part of this communication, vegetation was exccedingly rank and luxuriant, not only during the prevalence of the epidemic, but also antecedent to its visitation. Assuming, therefore, that certain degrees of heat and moisture, acting upon perishable matter, produce that state of atmosphere which is the predisposing cause of cholera, it will not be an easy matter to determine why one season should develope the disease, whilst another, precisely similar in all its characteristics, should be marked with a high degree of health, or, at least, free from any of those diseases approximating to cholera. In the description hereafter given of the locality of Chambersburg, mention will be made of *drains* and *sewers*, which might seem to act as auxiliary causes in the production of the disease—yet here the same difficulty meets us, for these constructions have been in existence for more than twenty-five years. In 1832, when the cholera first visited Chambersburg, this place was the great thoroughfare of travel between Philadelphia and Pittsburg, but since the opening of the Central railroad all that travel has been removed. The place may be regarded, therefore, as rather isolated in its situation, and enjoying for years a high character for health and salubrity.

These difficulties, which we encounter in endeavouring to discover any local agent, are calculated to dishearten the physician and to deter him from a close investigation. That it is a vitiated and poisonous state of the atmosphere which induces the disease there can be but little doubt, and that persons badly fed and meanly lodged, and those having debilitated constitutions, are more susceptible to the *poison* than those in vigorous health and living in ease and comfort, are also indisputable truths. But why this miasmatic atmosphere should affect in one place and not in another—should bring sickness and death in one particular season and not in another similar to it—should carry off one individual of a weak constitution and pass by his neighbor similarly affected—are questions to answer which we must acknowledge ourselves utterly incompetent. We must content ourselves with regarding them as among the great *arcana* of nature, the solution of which, perhaps, is reserved for future generations. The wisdom of Providence is manifested in his works, and may we not suppose that in this matter, as in many others, perfect *knowledge* would not contribute to our happiness. We daily see things around us which we do not comprehend, yet we doubt not of their existence, or of the laws by which they are governed. May it not be so with respect to various diseases which seize upon and affect the human system. The effect we see and know, but the cause lies beyond our vision and comprehension. It was remarked, during the prevalence of the great plague in London, that the weather was singularly fine. The sun shone with unusual brilliancy and splendor, whilst the atmosphere appeared pure and lucent. Yet who doubts that the seeds of infection and death were floating densely through the pestiferous air, invisible to mortal vision, and beyond all scientific investigation?

Before proceeding to the history of this epidemic, it may be proper to give a brief description of the locality of Chambersburg, and of its immediate neighborhood, so as to furnish data for those who may desire to speculate about the local causes which may have developed the disease; as well as afford information to others, desirous of investigating fully and satisfactorily, every thing connected with the existence and prevalence of this fearful malady.

Chambersburg, the judicial seat of Franklin County, Pennsylvania, is principally situated on the bank of the Conecocheague Creek (a small stream meandering southwardly into the Potomac,)

at the point where it receives the waters of the Falling Spring. The creek has its sources in the South Mountain—running about ten miles east of the town—which circumstance renders its waters somewhat turbid and discolored by the time they reach Chambersburg: whilst the Falling Spring, taking its rise from pure and clear springs, but a few miles from the town—pours its tribute into the Conecocheague almost as fresh and translucent as when it receives it from the fountain-head. A short distance below the junction of these streams and nearly opposite the centre of the town, a dam has been constructed for the purpose of carrying on "Lemno's Factory." About one fourth of a mile lower down, another dam has been formed to serve the purpose of a common grist mill. To the north of the town across the creek, and to the east upon the spring, many other *dams* have been erected for manufacturing purposes. The principal streets in Chambersburg run parallel with the creek. These are crossed at right angles by four or five others. From the natural situation of the place all these streets are admirably well drained. That part of the town, on the west bank of the creek, lies on the side of a gently sloping hill, becoming gradually more precipitous as you advance westwardly. The basis of this section is slate intermixed with tumbling stone. The water used for domestic purposes, in this neighborhood is soft and clear. On the eastern bank of the creek, where the main portion of the town is located, the soil is limestone, covered with a stiff, red clay, overlaid with a stratum of dark rich earth. There are no marshes or ponds, of any account in the vicinity. Great attention has been paid to grading the streets and alleys—and a sewer has been built, leading from Main street to the creek—a distance of 500 feet, which carries off all the filth and waste water from the eastern and central parts of the town. It must be remarked, however, that this beneficial construction emits, sometimes during the months of July and August, an unpleasant effluvium. Lime, during the past season, was plentifully distributed—as indeed it has been during the summer months for several years past.

I have been thus particular in these details, as, during the progress of my remarks, I shall occasionally allude to them. Besides, it is my design, in this communication, simply to state all the facts connected with the locality of the place and neighborhood—the incidents of the weather—heat and moisture, leaving it for those who are fond of investigating cause and effect to draw their own conclusions.

I now subjoin a tabular register of each day's observation, giving a condensed view of the greatest heat, with other meteorological notices, commencing with April and ending with September, 1852.

*April.* Six days, thermometer at and above 55°; highest 62°. Two slight snows in this month.

The whole of this month nearly either rainy or cloudy, average height of thermometer 50°.

*May.* Fourteen days, thermometer stood at 70° and upwards—highest 82°. From first to sixth, clear and windy; remainder of the month either cloudy or rainy.

*June.* Twelve days, thermometer stood at 80° and upwards—highest 90°. Seven days clear, remainder showery, cloudy, or windy.

*July.* Twenty-five days, thermometer stood at 80° and upwards—highest 98°. First, cloudy and showery. From this to the 13th no rain, but cloudy and windy. Balance of the month either cloudy or showery.

*August.* Nine days, thermometer stood at 80° and upwards—highest 86°. Clouds and showers characterized the greater part of this month. But four days that can properly be styled *clear*; average height of thermometer 76°.

*September.* Five days, thermometer at 80° and upwards, highest 84°. Fourteen days, thermometer at 70° and upwards—average 74°. A greater part of the month either cloudy or rainy—with more clear days, however, than in August.

It will be observed, by inspecting the foregoing register, that clouds and rain predominated during the past summer months, with a higher degree of heat, so that the season may emphatically be denominated a *wet* one. As a consequence vegetation of all kinds abounded. Indeed, the luxuriance and redundancy of the vegetable kingdom not only attracted the attention of close observers, but was a subject of universal observation. Even upon our poorest slate farms, where the crops are most easily affected by any derangement of the weather, it was remarked that all the various crops were unusually abundant. It will also be observed, that a drought of thirteen days preceded the first appearance of the cholera, though a heavy rain fell a day or two immediately before its breaking out on the 15th July.

The first case of the epidemic made its appearance in Main street, near the centre of the town, and about twenty paces from the entrance of the public sewer. It occurred on the fifteenth of July, and, what is singular, in the very room in which it made its appearance in 1832. Both the cases proved fatal. During this visitation the patient was seized in the early part of the evening with diarrhœa, for which his family physician prescribed. About the turn of the night, vomiting came on, and by four o'clock in morning he was collapsed. His appearance was now entirely changed, presenting a livid hue, with a cold clammy skin, and a feeble pulse; at noon he died. On the evening of the same day a lady in the adjoining house was taken with diarrhœa. About four o'clock in the morning I was called to see her. I found her pulse nearly extinct, with a cold clammy feeling of the skin, her lips livid, tongue cold, eyes sunk deep in their sockets, in short her whole system was shrunken and cadaverous. She was bathed in a cold perspiration, her fingers shrivelled, "white and corrugated like those of a washerwoman after a hard day's work." Her voice was husky, but her mind sound and unimpaired midst these ruins of organic life. She continued in this state until evening, when she expired.

The next case occurred on the 26th. This was that of an old man, yet he lingered several hours longer than either of those I have described. From this date until the 31st, no deaths occurred. On the latter day there were five deaths from cholera, all differing in complexion. Two of these cases were stout, healthy and temperate men—one, a widowed lady living retired and in easy circumstances—one, a young colored girl, and an intemperate colored man. One of these was distinguished by the length of time the cramps and pains continued. Up to a short time before her death, she gave utterance to violent screams, attesting her great agony and suffering. From the 2d until the 12th of August sixteen deaths were registered—all of this disease, varying somewhat in character and duration. A remark may here be made as to these sixteen cases, and applied to any period throughout the continuance of the epidemic, viz: That the fatal pest seemed to attack at random—bringing both young and old, temperate and intemperate—the man enjoying apparently vigorous health, and him of a weakly constitution, all under its baneful influence. From the 12th to the 20th there were eleven cases. Most of these were colored persons, and some of very loose habits.

From this date until the 30th, a suspension of the epidemic took place. No case occurred, and the citizens were flattering themselves that the danger was passed, and were congratulating each other upon their escape from its ravages, when suddenly, upon the 30th, it re-appeared, and continued its course with recruited vigor. During the month of September there were thirty-five deaths. It was in this month that the epidemic assumed its most aggravated form. The cases generally were more rapid, terminating in from six to eight hours. In one case that occurred on the 17th, death ensued in five hours from the time of attack. In another case, that of a stout and healthy man, who died in six hours after being attacked, I observed a peculiar twitching and muscular motion of the arms and legs, about thirty minutes after he had ceased to breathe. The urinary secretion in nearly all of the cases was diminished, and in many entirely suppressed. Symptoms of greater gravity characterized the disease as it progressed. The unmistakeable cholera countenance made its appearance simultaneously with the attack. The eyes at once became sunken in their orbits, blood-shotten, and surrounded by a black circle. The lips were livid—cramps of the extremities and abdomen, so harrowing in some of the first cases, became slight and feeble. So powerful was the poison that the system was at once depressed beyond reaction. The respiration was quick and difficult—pulse rapid—varying from 100 to 120. In some few cases there was an apparent suspension, which, however, lasted for a short time only. In the course of a few hours, the disease resumed its fatal progression. With the small number of patients who recovered after collapse had taken place, the symptons continued gradually to improve. The respiration became slow, the pulse feeble and slow, and the thirst less intense. The convalescence, however, was tedious, requiring several days before they could leave their beds.

Several cases occurred during the month of October, but at longer intervals. The weather, however, was unusually warm for the season, and it was not until the frosts of November made their appearance that it entirely disappeared.

In giving this account of the epidemic, I have confined myself to the town of Chambersburg. Many cases occurred in the adjoining districts, some of which I attended; but I have not thought proper to connect their history with those of this place, though I have included them in giving the gross number ef deaths.

After having described the locality of the operation of this erratic pest—given an account of the state of the weather antecedent to its appearance and during its prevalence—together with a history of the cases, it may not be amiss to make a few remarks relative to the *treatment* pursued. And here I find myself at a loss, for I can necessarily only speak of my own mode of procedure. I consulted, however, with some of my brother physicians, and I am happy to state that I found their observations, experience, and manner of treatment, to coincide generally with my own.

As it has already been remarked, cholera presents several stages in its progress, The various phenomena of the disease had to be encountered with various remedies, adapted to that particular stage. If called in when the first symptoms were prevailing, the grand object was to arrest the diarrhœa. The agents used for this purpose were opium and its preparations, sugar of lead and other powerful astringents. The onset of the attack, generally, was easily checked, and, if prudently watched, its further progress was prevented. Many, however, were not alarmed by the simple alvine evacuations, particularly when unaccompanied by pain, and thus carelessly suffered the insidious foe to enter the citadel, before aroused to a true sense of their danger. In most cases that I attended, such was the case; diarrhœa had been permitted to continue—sometimes for days—before they thought of calling in medical aid.

During the continuance of the diarrhœa, purgatives of any kind, even laxatives, were found to aggravate the disease. Calomel, which has been recommended by so many writers as a *Sampsonian* remedy, I found invariably to accelerate the collapse, even when administered in the minutest portions. When

I found the patient approximating to this fatal stage of the disease, and when actually fallen into it, recourse was had to still more active remedies. In addition to the medicines already mentioned, (which were continued throughout the entire course,) recourse was had to ammonia, camphor, brandy, wine and other stimulants, to sustain the sinking powers of the system. Hot outward applications, sinapisms to the stomach and extremities, stimulating liniments, accompanied with friction, were added to the number of remedial agents. If, happily, this active manner of treatment succeeded in arousing the collapsed patient and arresting its further progress, then small doses of calomel or blue mass, combined with small portions of opium to restrain its operation, were found serviceable in restoring the liver to its proper functions. The greatest care, however, had to be given to their diet, for the slightest deviation from the strictest rule, invariably endangered a relapse. But the inveterate nature of the disease most frequently thwarted the most anxious attention and watchful care, and when the too sanguine physician imagined that he had succeeded in effecting a cure, he suddenly found himself deprived of the object of his deepest solicitude. The fell monster seemed to take delight in baffling the most active efforts and eager expectations, and moved from victim to victim with a tenacity of purpose equalled only by his almost unrivalled success.

*Remarks.*—The symptoms of all these cases were identical in character with those of the disease in this country in 1832. The most perceptible difference between the cases which I have mentioned, and those which occurred during its first visitation, consisted in the latter being more aggravated and fatal. In most cases during the present epidemic, a few hours sufficed to determine the fate of the victim. So rapid and vigorous were the attacks of the disease in many cases, that, notwithstanding the general alarm upon the subject—and the eager anxiety to procure medical aid as speedily as possible, frequently before assistance could arrive, the first stage of collapse had taken place. In a few cases that I attended, even when called to the patient when first attacked, the disease continued to progress with such frightful rapidity that the fatal collapse stared you in the face in the midst of all the appliances and medical means which had proved beneficial to others. My experience corroborates what has been remarked by nearly every medical writer upon this subject, viz: that when the patient once passes into the state of collapse, very little can be done for him. Death almost universally ensues.

The unmistakeable identity which connects the cases of the past season with those of 1832, leads us to inquire respecting the origin of this disease, and why the atmosphere of Chambersburg should, apparently, present a congenial field for its operations. This question affords ample scope for philosophical investigation. I shall confine myself, as already intimated, to a simple statement of facts.

The first cases of cholera in 1832 occurred immediately after a heavy fall of rain, which had been preceded by several weeks of dry weather. It will be seen by reference to the accompanying register that the appearance of this disease on the 15th of July last was ushered in by a copious shower of rain. During the two previous weeks the weather was very dry and hot. The same remark is applicable to its intermission and re-appearance during August and September. On the 18th and 19th of October, some cases occurred, all of which proved fatal. From this until the 30th, the weather was dry, warm and clear. On that day it rained heavily, and on the 31st, two cases of cholera made their appearance, which, however, yielded to medicine.

Whether these changes in the atmosphere had anything to do in developing the disease, or were simply remarkable coincidences, I will not pretend to decide, leaving it for those more conversant with atmospheric mutations to form an opinion. I am inclined, however, to believe that a vitiated air, acted upon by some sudden and exciting cause, is the principal agent in the production of certain diseases and their concomitant consequences.

In view, then, of all the facts and circumstances connected with the existence of the late epidemic in Chambersburg, must we not regard it as somewhat remarkable that a town, hitherto celebrated for the high degree of health enjoyed by its inhabitants, should suddenly, after an interval of twenty years, be visited by a scourge so formidable and deadly in its character? A town, too, in which the civil authorities had taken the precaution to have all the streets and alleys thoroughly cleaned and limed, in which an unusual degree of care was bestowed upon the dwellings of the citizens, and the quality and kind of their food—should be so severely attacked and for such a length of time, is one of those mysterious movements in nature for which which it is impossible to account. More than one hundred and thirty persons were swept away in the town and neighborhood by this terrible pestilence, without any perceptible difference between this season and many preceding ones, and, with the exception of the town of Mifflin, not another place in the State was similarly affected.

